# Testing novel strategies for patients hospitalised with HIV-associated disseminated tuberculosis (NewStrat-TB): protocol for a randomised controlled trial

**DOI:** 10.1186/s13063-024-08119-4

**Published:** 2024-05-08

**Authors:** Phiona E. Namale, Linda Boloko, Marcia Vermeulen, Kate A. Haigh, Fortuna Bagula, Alexis Maseko, Bianca Sossen, Scott Lee-Jones, Yoliswa Msomi, Helen McIlleron, Ayanda Trevor Mnguni, Thomas Crede, Patryk Szymanski, Jonathan Naude, Sakeena Ebrahim, Yakoob Vallie, Muhammed Shiraz Moosa, Ismail Bandeker, Shakeel Hoosain, Mark P. Nicol, Nazlee Samodien, Chad Centner, Wentzel Dowling, Paolo Denti, Freedom Gumedze, Francesca Little, Arifa Parker, Brendon Price, Denzil Schietekat, Bryony Simmons, Andrew Hill, Robert J. Wilkinson, Ida Oliphant, Siphokazi Hlungulu, Ivy Apolisi, Monica Toleni, Zimkhitha Asare, Mkanyiseli Kenneth Mpalali, Erica Boshoff, Denise Prinsloo, Francisco Lakay, Abulele Bekiswa, Amanda Jackson, Ashleigh Barnes, Ryan Johnson, Sean Wasserman, Gary Maartens, David Barr, Charlotte Schutz, Graeme Meintjes

**Affiliations:** 1 Wellcome Centre for Infectious Diseases Research in Africa (CIDRI-Africa), Institute of Infectious Disease and Molecular Medicine, University of Cape Town, Cape Town, South Africa; 2 Department of Medicine, University of Cape Town, Cape Town, South Africa; 3 Institute of Infection, Veterinary and Ecological Sciences, University of Liverpool, Liverpool, UK; 4 Division of Clinical Pharmacology, Department of Medicine, University of Cape Town, Cape Town, South Africa; 5 Department of Medicine, Khayelitsha Hospital, Cape Town, South Africa; 6 Department of Medicine, Stellenbosch University, Stellenbosch, South Africa; 7 Department of Medicine, Mitchells Plain Hospital, Cape Town, South Africa; 8 Division of Medical Microbiology, Department of Pathology, University of Cape Town, Cape Town, South Africa; 9 Division of Infection and Immunity School of Biomedical Sciences, University of Western Australia, Perth, Australia; 10 Department of Statistical Sciences, University of Cape Town, Cape Town, South Africa; 11 Division of Anatomical Pathology, Department of Pathology, University of Cape Town, Cape Town, South Africa; 12 LSE Health, London School of Economics and Political Science, London, UK; 13 Francis Crick Institute, London, UK; 14 Department of Medicine, Imperial College London, London, UK; 15 Department of Medicine, New Somerset Hospital, Cape Town, South Africa

**Keywords:** HIV, Disseminated tuberculosis, High dose rifampicin, Levofloxacin, Prednisone, Randomised controlled trial

## Abstract

**Background:**

HIV-associated tuberculosis (TB) contributes disproportionately to global tuberculosis mortality. Patients hospitalised at the time of the diagnosis of HIV-associated disseminated TB are typically severely ill and have a high mortality risk despite initiation of tuberculosis treatment. The objective of the study is to assess the safety and efficacy of both intensified TB treatment (high dose rifampicin plus levofloxacin) and immunomodulation with corticosteroids as interventions to reduce early mortality in hospitalised patients with HIV-associated disseminated TB.

**Methods:**

This is a phase III randomised controlled superiority trial, evaluating two interventions in a 2 × 2 factorial design: (1) high dose rifampicin (35 mg/kg/day) plus levofloxacin added to standard TB treatment for the first 14 days versus standard tuberculosis treatment and (2) adjunctive corticosteroids (prednisone 1.5 mg/kg/day) versus identical placebo for the first 14 days of TB treatment. The study population is HIV-positive patients diagnosed with disseminated TB (defined as being positive by at least one of the following assays: urine Alere LAM, urine Xpert MTB/RIF Ultra or blood Xpert MTB/RIF Ultra) during a hospital admission. The primary endpoint is all-cause mortality at 12 weeks comparing, first, patients receiving intensified TB treatment to standard of care and, second, patients receiving corticosteroids to those receiving placebo. Analysis of the primary endpoint will be by intention to treat. Secondary endpoints include all-cause mortality at 2 and 24 weeks. Safety and tolerability endpoints include hepatoxicity evaluations and corticosteroid-related adverse events.

**Discussion:**

Disseminated TB is characterised by a high mycobacterial load and patients are often critically ill at presentation, with features of sepsis, which carries a high mortality risk. Interventions that reduce this high mycobacterial load or modulate associated immune activation could potentially reduce mortality. If found to be safe and effective, the interventions being evaluated in this trial could be easily implemented in clinical practice.

**Trial registration:**

ClinicalTrials.gov NCT04951986. Registered on 7 July 2021

https://clinicaltrials.gov/study/NCT04951986

**Supplementary Information:**

The online version contains supplementary material available at 10.1186/s13063-024-08119-4.

## Administrative information

**Table:** 

**Title** {1}	Testing Novel Strategies for Patients Hospitalized with HIV-associated Disseminated Tuberculosis (NewStrat-TB): Protocol for a Randomised Controlled Trial
**Trial registration** {2a and 2b}	This trial is registered with Clinical Trials.gov NCT04951986 and the South African National Clinical Trials Register (SANCTR) DOH-27072021-4806.
**Protocol version** {3}	1.5, 22 February 2023
**Funding** {4}	The trial is funded by a Wellcome Investigator Award (214321/Z/18/Z) with additional support from the Wellcome Centre for Infectious Diseases Research in Africa (CIDRI-Africa, 203135/Z/16/Z) and funding from the South African Research Chairs Initiative of the Department of Science and Technology and National Research Foundation (NRF) of South Africa (Grant No 64787). KAH is funded by a Wellcome Clinical PhD Fellowship (University of Liverpool block award grant number 203919/Z/16/Z). The funders have no role in the study design, data collection, data analysis, data interpretation, or writing of this manuscript. The trial received additional funds from the South African Medical Research Council Institutional Researcher Programme, under the Research Capacity Development (RCD) Scholarship.
**Author details** {5a}	1. Wellcome Centre for Infectious Diseases Research in Africa, Institute of Infectious Disease and Molecular Medicine, University of Cape Town, South Africa 2. Department of Medicine, University of Cape Town, South Africa 3. Institute of Infection, Veterinary and Ecological Sciences, University of Liverpool, United Kingdom 4. Division of Clinical Pharmacology, Department of Medicine, University of Cape Town, South Africa 5. Department of Medicine, Khayelitsha Hospital, South Africa 6. Department of Medicine, Stellenbosch University, South Africa 7. Department of Medicine, Mitchells Plain Hospital, South Africa 8. Division of Medical Microbiology, Department of Pathology, University of Cape Town, South Africa 9. Division of Infection and Immunity School of Biomedical Sciences, University of Western Australia, Australia 10. Department of Statistical Sciences, University of Cape Town, South Africa 11. Division of Anatomical Pathology, Department of Pathology, University of Cape Town, South Africa 12. LSE Health, London School of Economics and Political Science, London, United Kingdom 13. Francis Crick Institute, London, United Kingdom 14. Department of Medicine, Imperial College London, London, United Kingdom 15. Department of Medicine, New Somerset Hospital, South Africa
**Name and contact information for the trial sponsor** {5b}	University of Cape Town, Private Bag X3, Rondebosch 7701, South Africa +27 (0)21 406 6340 +27 (0)21 650 3002 https://www.uct.ac.za
**Role of sponsor** {5c}	This is an investigator-initiated study. The sponsor plays no role in study design; data collection, management, analysis, or interpretation; writing of the report; or the decision to submit the report for publication.

## Introduction

### Background and rationale {6a}

Tuberculosis (TB) is the leading cause of death (40%), hospitalisation (18%) and in-hospital death (25%) in people living with the human immunodeficiency virus (HIV) globally [1]. Case fatality rates of hospitalised patients diagnosed with HIV-associated TB in Africa range between 11 and 32% [2–6]. A study conducted in Malawi and South Africa nested within the STAMP trial [7] described clinical characteristics and mortality in HIV-positive patients who had rapid urine-based screening for TB upon admission to hospital [6]. Mortality was 30.7% by day 56 in patients with confirmed TB by Xpert MTB/RIF^Ⓡ^ (Cepheid) or urine Determine TB-lipoarabinomannan (LAM) Antigen^Ⓡ^ (Abbott) assay. The median time to death was 12 days (interquartile range, IQR 5–27 days) and 32.3% of deaths occurred within 7 days of admission [6].

Our research group previously reported 12-week mortality of 21.5% in an observational cohort study of 576 HIV-positive patients diagnosed with TB whilst admitted to Khayelitsha Hospital, South Africa [4]. Over half of the deaths in this study occurred in the first 14 days after enrolment and 37% of deaths occurred within 7 days despite timeous initiation of anti-tuberculosis treatment [4]. Key findings from this study were that mortality was associated with high blood concentrations of host biomarkers characterising sepsis, higher number of positive markers of TB dissemination (urine Alere LAM, urine Xpert MTB/RIF, blood Xpert MTB/RIF Ultra and tuberculosis blood culture) [4] and an immune signature characterised by higher blood concentrations of soluble mediators associated with the innate immune system [4]. Amongst the biochemical markers of sepsis, patients who died had significantly higher venous lactate, C-reactive protein, procalcitonin, D-dimer and creatinine.

Nested within this study [4] was a sub-study that assessed Xpert MTB/RIF Ultra (Xpert Ultra) as a diagnostic tool for TB blood stream infection and investigated cycle threshold as a quantitative disease biomarker [8]. Blood Xpert Ultra was positive in 37% of patients (165 of 447) with microbiologically confirmed tuberculosis [8]. Quantitative blood Xpert Ultra results (cycle threshold) were more closely associated with mortality than other TB biomarkers including blood culture, urine LAM or urine Xpert [8].

A meta-analysis has shown that disseminated TB (mycobacterial spread through the blood stream to organs such as the liver, spleen and lymph nodes) was found in 88% of post-mortems of HIV-positive adults who died with active TB [7]. Our research group has explored the contribution of disseminated TB to mortality using three tests where positivity is associated with TB dissemination: urine Alere LAM (Alere) ≥1 reading, urine Xpert MTB/RIF positive for *Mycobacterium tuberculosis* (MTB), and Myco F/lytic blood culture positive and MTB identified [4]. In the Khayelitsha observational cohort 65% of patients hospitalised with HIV-associated TB had evidence of disseminated TB defined as one or more of these markers being positive. Positivity of these markers was associated with death and patients with a higher number of positive biomarkers for TB dissemination had higher mortality [4]. Blood cultures were positive for MTB in 38.2% of patients overall and in 51.6% of patients who died. A sub-study quantified mycobacterial load in blood and changes over the first 72 h of anti-tuberculosis treatment using three tests: Xpert Ultra, Myco/F lytic culture and a novel microscopy technique using a fluorescent probe 4-N,N-dimethylaminonaphthalimide-trehalose (DMN-Tre). Patients who died had a higher probability of having a higher mycobacterial load at all time points compared to survivors and showed some evidence of slower clearance of these markers on anti-tuberculosis treatment [9].

Furthermore, in the Khayelitsha observational study, soluble mediators of inflammation were measured, and two major immune profiles were identified using principal components analysis. Patients who died had higher concentrations of soluble mediators related to the innate immune system and chemotactic signalling such as IL-8, MIP-1β/CCL4, IP10/CXCL10 and MIP-1α, the anti-inflammatory mediator, IL-1Ra and the pro-inflammatory marker IL-6. These mediators dominated one of the two major principal components and was associated with mortality when included in a Cox proportional hazards model (adjusted hazards ratio (aHR) =2.2 95% confidence interval (95% CI) = 1.9–2.7) after adjusting for age, HIV viral load and sex. Mediators broadly classified as T-cell associated (IL-4, IL-17, RANTES/CCL5, IL-7, IL-12p70, IL-5, IFN-γ, IL-13 and growth factors (fibroblast growth factor, platelet-derived growth factor and transforming growth factor-beta 1) were lower in patients who died and dominated the second and third principal component. This profile was protective when included in a Cox proportional hazards model with aHR = 0.7 (95% CI = 0.5–1.0) [5].

International treatment guidelines currently recommend that hospitalised HIV-positive patients with disseminated TB who often present with clinical features compatible with a sepsis syndrome [10, 11] are treated with the same initial treatment used for ambulant HIV-negative outpatients with pulmonary tuberculosis. Based on our prior research, we think that disseminated HIV-associated TB is a more severe clinical entity than pulmonary TB with much higher risk of fatal outcomes. Therefore, specific therapeutic interventions need to be developed and evaluated for treatment of this condition. Intensified anti-TB treatment that reduces the highly disseminated mycobacterial load more rapidly in these patients could potentially improve clinical outcomes. Immunomodulation of aberrant host responses could also potentially improve survival. This clinical trial evaluates two novel treatment strategies (intensified initial tuberculosis treatment and immunomodulation with corticosteroids) for patients hospitalised with HIV-associated disseminated TB with the aim of informing treatment guidelines and improving outcomes for this patient population.

### Rationale for trial interventions

1. Intensified tuberculosis treatment

The first intervention being evaluated is intensified initial tuberculosis treatment which includes both higher dose rifampicin (35 mg/kg/day) and the addition of levofloxacin to the initial 14 days of standard tuberculosis treatment to potentially increase bactericidal activity of the regimen and more rapidly reduce the disseminated mycobacterial load.

- Higher dose rifampicin

Rifampicin was introduced as a tuberculosis treatment in the 1970s and was initially limited to a maximum dose of 10 mg/kg/day mainly due to fear of dose-related hepatic toxicity but also due to cost considerations [12]. Recent studies suggest that this dose does not achieve optimal antimicrobial activity and doses of up to 50 mg/kg have been tested in clinical trials. The PanACEA MAMS-TB trial was a randomised controlled open label trial that investigated different rifampicin doses in combination with other standard first-line tuberculosis drugs and regimens where standard drugs were replaced by moxifloxacin or SQ109 in patients with pulmonary tuberculosis [13]. The primary endpoint was time to sputum culture conversion in liquid media. The regimen including rifampicin 35mg/kg/day resulted in significantly faster sputum conversion in liquid culture by 12 weeks. Patients in the rifampicin 35 mg/kg/day arm achieved culture conversion in liquid media at a median of 48 days compared to 62 days in the control arm with rifampicin 10 mg/kg/day (adjusted HR 1.78, 95% CI 1.22–2.58) [13].

The safety profile of the experimental arm with rifampicin 35 mg/kg/day in the PanACEA MAMS-TB trial was similar to the control arm with rifampicin 10 mg/kg/day [13]. A small dose finding trial that enrolled 60 patients with tuberculous meningitis in Indonesia suggested improved survival in these patients when using high dose rifampicin (30 mg/kg/day) [14].

The PanACEA HIGHRIF1 was a study to evaluate the safety, tolerability, pharmacokinetics and early bactericidal activity of higher doses of rifampicin. Patients received 40 or 50 mg/kg rifampicin once daily as monotherapy (days 1–7), thereafter supplemented by standard doses of other TB medication namely isoniazid, pyrazinamide and ethambutol (days 8–14). During monotherapy, 87% (13 out of 15) of patients in the 40 mg/kg dose group reported 36 adverse events in total whilst all 17 patients in the 50 mg/kg dose reported a total of 93 adverse events [15]. The adverse events were mostly mild or moderate and related to tolerability rather than safety. These included gastrointestinal disorders, pruritis, hyperbilirubinemia and jaundice [15]. Together, these studies suggest that doses of rifampicin up to 40 mg/kg/day, but not higher, are safe and tolerated.

Higher doses of rifampicin result in non-linear increases in plasma exposure due to saturation of hepatic metabolism. Pharmacokinetic models which incorporate autoinduction and saturation of hepatic enzymes have shown that increasing the rifampicin dose to 35 mg/kg/day results in 667% higher predicted 24-h plasma area under the curve (AUC) at 14 days compared to 10 mg/kg/day [16] with >99% of patients attaining target exposure in plasma defined by murine models, compared to 60% of patients who received rifampicin 10 mg/kg/day [17, 18].

b) Addition of levofloxacin

Fluoroquinolones have been used in the treatment of tuberculosis since the 1980s when ofloxacin was used to treat patients who had failed first-line treatment, including patients with drug resistance [19]. In vitro and animal studies have showed that fluoroquinolones have potent bactericidal and sterilising activity [20–22]. Human studies have demonstrated early bactericidal activity (EBA) for moxifloxacin that approximates isoniazid. EBA is defined as the fall in the log [10] colony-forming units (cfu) of *Mycobacterium tuberculosis* per millilitre of sputum per day. The mean EBA for moxifloxacin in one study was 0.53 (95% CI 0.28–0.59) and that for isoniazid 0.77 (95% CI 0.54–1.00) [23].

The efficacy of fluoroquinolones has been studied extensively in ‘TB treatment-shortening’ clinical trials involving the replacement of an individual drug in the standard first-line regimen with a fluoroquinolone and a shorter duration of the regimen [24–26]. Two studies used moxifloxacin [25, 27], whilst one used gatifloxacin [24]. These studies did not demonstrate non-inferiority to standard therapy in terms of successful end-of-treatment outcomes. However, patients receiving fluoroquinolones demonstrated a shorter time to sputum culture-conversion compared to standard therapy [25]. In vitro studies have shown that moxifloxacin is the most active fluoroquinolone against rapidly growing *Mycobacterium tuberculosis* with minimum inhibitory concentrations (MIC) comparable to that of isoniazid [28, 29].

In an open-label phase 3 randomised controlled trial comparing 4 months rifapentine containing regimens with or without moxifloxacin for drug-sensitive pulmonary tuberculosis, the regimen containing moxifloxacin was non-inferior to the standard TB regimen (control) [30]. Participants in the 2 intervention arms received 8 weeks of rifapentine (1200 mg), isoniazid and pyrazinamide at the standard doses and either ethambutol (standard doses) or moxifloxacin at 400 mg daily. The 9-week continuation phase for both these arms did not include pyrazinamide whilst the group without moxifloxacin also dropped ethambutol after the first 8 weeks. The control regimen consisted of standard TB treatment with rifampicin, isoniazid, ethambutol and pyrazinamide for 8 weeks and 18 weeks of rifampicin and isoniazid [30]. The addition of rifapentine at high daily doses with moxifloxacin showed evidence for treatment shortening for tuberculosis [30].

There are drug–drug interactions between moxifloxacin and rifampicin that resulted in reduced moxifloxacin exposure [31, 32]. In a study by Nijland and colleagues, the moxifloxacin mean AUC (0–24 h) was reduced by 31% and mean maximum concentration (Cmax) by 32% when co-administered with rifampicin in healthy volunteers [31]. Levofloxacin does not have drug–drug interactions with rifampicin and is less likely to cause QT prolongation than moxifloxacin [33] and was therefore chosen as the fluoroquinolone in this trial.

Levofloxacin’s bactericidal activity is concentration dependent, and efficacy is predicted by AUC (0–24 h) to minimum inhibitory concentration (MIC) ratio. Levofloxacin has excellent early bactericidal activity (EBA) against *Mycobacterium tuberculosis* and can be administered as a once-daily dose. When isoniazid and levofloxacin were given as monotherapy for patients with sputum-positive TB for 7 days, levofloxacin had a comparable mean EBA in the first 2 days (0–2) of 0.45 log 10 cfu/ml/day (SD 0.35 and 95% CI 0.20–0.71) to that of isoniazid which was 0.67 log10 cfu/ml/day (SD 0.17 and 95% CI 0.55–0.80). Side effects of fluoroquinolones include headache, fever, rash, vomiting, diarrhoea, visual disturbance and tenderness over tendons. The more severe adverse events of fluoroquinolones include seizures and collagen-associated adverse events for example aortic aneurysms, dissections and tendon ruptures [34, 35]. The use of levofloxacin requires a risk–benefit consideration, but severe side effects are rare. Levofloxacin also has activity against community acquired gram-negative and gram-positive bacteria, another potential benefit. We hypothesise that the addition of levofloxacin and higher dose rifampicin will increase the bactericidal efficacy of the regimen, reduce disseminated *Mycobacterium tuberculosis* load more rapidly and improve survival in this patient population.

2) Adjunctive corticosteroids for immune modulation

The second intervention being evaluated is adjunctive prednisone, a corticosteroid, administered together with the initial 14 days of tuberculosis treatment. Corticosteroids are commonly used as adjunctive therapy in the treatment of infections for their immune modulatory properties. Corticosteroid adjunctive therapy reduced mortality in TB meningitis, constriction in TB pericarditis and hospitalisation when used for treatment of TB-IRIS (immune reconstitution inflammatory syndrome) [36–39]. In a randomised controlled trial for the prevention of paradoxical TB-IRIS conducted by our group, prednisone reduced incidence of TB-IRIS by 30% [40]. Meta-analyses show that adjunctive corticosteroids reduce mortality in pneumonia (RR = 0.66, 95%CI = 0.47–0.92) and sepsis (RR = 0.87, 95%CI = 0.76–1.00) [41, 42].

In a phase 3 multicentre, randomised controlled trial amongst intensive care unit patients with severe community-acquired pneumonia receiving standard therapy including antibiotics and supportive care, hydrocortisone 200 mg daily for either 4 or 8 days as determined by clinical improvement and then tapering for 8 or 14 days reduced the risk of death by day 28 [43]. Amongst 795 patients whose data was analysed, death by day 28 had occurred in 6.2% (CI 3.9–8.6) in the hydrocortisone arm compared to 11.9% (CI −8.7–15.1) in the placebo arm [43].

The potential side effects of prednisone include gastritis, hyperglycaemia, hypertension, avascular necrosis, myopathy, depression, mania, increased risk of malignancy and infections ([44, 45]. In the PredART trial investigating prednisone for the prevention of paradoxical TB-associated IRIS, there was no difference in the incidence of new AIDS (acquired immune deficiency syndrome) defining illnesses or invasive bacterial infections in the prednisone group compared to placebo. There were no differences in glucocorticoid side effects between the prednisone and placebo group [40]. In the investigation of the management of tuberculous pericarditis (IMPI) trial evaluating prednisolone and *Mycobacterium indicus pranii*, prednisolone was given for 6 weeks (120 mg for a week, then 30 mg reduction per subsequent week until 30 mg in week 4 then 15 mg and 5 mg in the last week). There was no difference in the incidence of opportunistic infections in the prednisolone group compared to the placebo group, 6.89/100 patient years and 5.91/100 patient years, respectively, HR = 1.16 (95% CI 0.84–1.61) [36]. Prednisolone was however associated with a higher incidence of cancers in HIV-positive patients [36].

A Cochrane systematic review assessed the effects of adjunctive corticosteroids on overall mortality and the need for mechanical ventilation in HIV-positive patients with pneumocystis pneumonia and substantial hypoxemia [46]. The review included studies that compared corticosteroids to placebo or usual care in addition to baseline treatment with cotrimoxazole, pentamidine or dapsone-trimethoprim and reported mortality data [46]. The risk ratios for overall mortality for adjunctive corticosteroids were 0.56 (95% CI 0.32–0.98) at 1 month and 0.59 (95% CI 0.41–0.85) at 3–4 months follow-up. Amongst adults, nine patients needed to be treated to prevent one death in a setting without antiretroviral therapy (ART) [46]. Adjunctive corticosteroids were not associated with an increase in severe opportunistic complications except for oesophageal candidiasis in a study by Gallant and colleagues [46]. One study by Bozzette and colleagues [47] found an increase in mucocutaneous herpes simplex infections. Pneumocystis pneumonia is more common with CD4 counts below 200 cells/mm^3^ and in patients not on ART [48]. Mortality benefit from corticosteroids was demonstrated in this group of patients with severe immunosuppression from advanced HIV without an increased incidence of major corticosteroid side effects. The increased risk of infections is a concern in patients with disseminated HIV-associated TB patients, but the evidence above does not suggest an increased risk of life-threatening infections when courses of prednisone of less than 4 weeks duration have been used.

In the prior observational cohort study of patients hospitalised with HIV-associated tuberculosis conducted by our group, mortality was associated with elevated plasma concentrations of soluble inflammatory mediators, especially innate immune mediators and lower concentrations of lymphocyte-associated mediators [4]. Corticosteroids modulate the innate immune response and inhibit inflammatory responses by regulating gene expression through interaction with the glucocorticoid response element; they also cause direct protein–protein interference with transcription and affect cellular signal transduction cascades involving secondary messengers [49].

The use of corticosteroids in TB of various clinical phenotypes has been studied for decades, yet no controlled evidence exists for their use in patients with disseminated tuberculosis [49, 50]. One rationale for the use of adjunctive corticosteroids in disseminated tuberculosis is that tuberculosis treatment may result in transient inflammation-mediated deterioration provoked by bacillary death and antigen release. In one study, HIV-negative patients with severe pulmonary tuberculosis had an increase in TNF-a and lactate during early treatment [51]. Our finding in the prior observational cohort study [4] that mortality was associated with higher concentrations of markers of innate immune activation and chemotactic signalling combined with the efficacy and safety of corticosteroids in treating and preventing paradoxical TB-IRIS [40], which is an inflammatory condition characterised in part by innate immune activation, provides rationale for evaluating corticosteroids as adjunctive treatment in disseminated HIV-associated TB.

Severe and fatal SARS-CoV-2 (severe acute respiratory syndrome coronavirus 2) have been associated with higher concentrations of innate immune mediators in plasma [52, 53] similar to disseminated HIV-associated tuberculosis [4]. In a randomised controlled trial, dexamethasone at a dose of 6 mg daily for up to 10 days in hospitalised patients with SARS-CoV-2 infection requiring oxygen therapy resulted in improved 28-day survival [54]. The 28-day mortality rate ratio (RR) comparing the dexamethasone group to the standard of care group was 0.83 (95% CI 0.75–0.93). Amongst patients requiring mechanical ventilation and those who received oxygen therapy but no ventilation, the RR was 0.64 (95% CI 0.51–0.81) and RR = 0.82 (95%CI 0.72–0.94), respectively [54]. This provides further evidence that corticosteroids improve survival in other infections in which the host inflammatory response contributes to pathogenesis and outcome.

The use of prednisone or placebo as study medication in the NewStrat-TB trial will not overlap with the use of prednisone for the prevention of paradoxical TB-IRIS because patients not on ART will only start or restart ART after 2 weeks of tuberculosis treatment and would then start prophylactic prednisone at this time and after completing their study medication [54].

We are evaluating prednisone at a dose of 1.5 mg/kg/day for 14 days in a placebo-controlled strategy. Our hypothesis is that prednisone will favourably modulate the innate immune response and thereby reduce mortality in patients with disseminated HIV-associated tuberculosis.

3) Rationale for factorial design

The advantage of the 2 × 2 factorial design is its efficiency allowing investigators to evaluate two interventions in one trial, each of which is applicable to and has equipoise in the same patient population. Given the costs, time and effort involved in conducting large clinical trials in this complex patient population, there is strong rationale for addressing the efficacy and safety of both these interventions within the one trial, as opposed to undertaking two separate trials which would double the number of participants required and increase costs and time to results.

### Objectives {7}

The main objective of the study is to assess the safety and efficacy of both intensified TB treatment (addition of high dose rifampicin (35 mg/kg/day) plus levofloxacin to standard anti-tuberculosis therapy for initial 14 days) and immunomodulation with adjunctive prednisone for initial 14 days as interventions to reduce mortality in hospitalised patients with disseminated HIV-associated tuberculosis compared to standard anti-tuberculosis therapy.

The primary efficacy endpoint is all-cause mortality at 12 weeks.

The secondary objectives involve assessing the following:

1. In-hospital mortality during index admission.
2. All-cause mortality at 2 and 24 weeks, respectively.
3. Change in venous lactate over 14 days of study medication administration.
4. Change in C-reactive protein over 14 days of study medication administration.
5. Change in haemoglobin over 14 days of study medication administration.

The safety and tolerability of both interventions will also be assessed.

### Trial design {8}

**Fig. 1:**
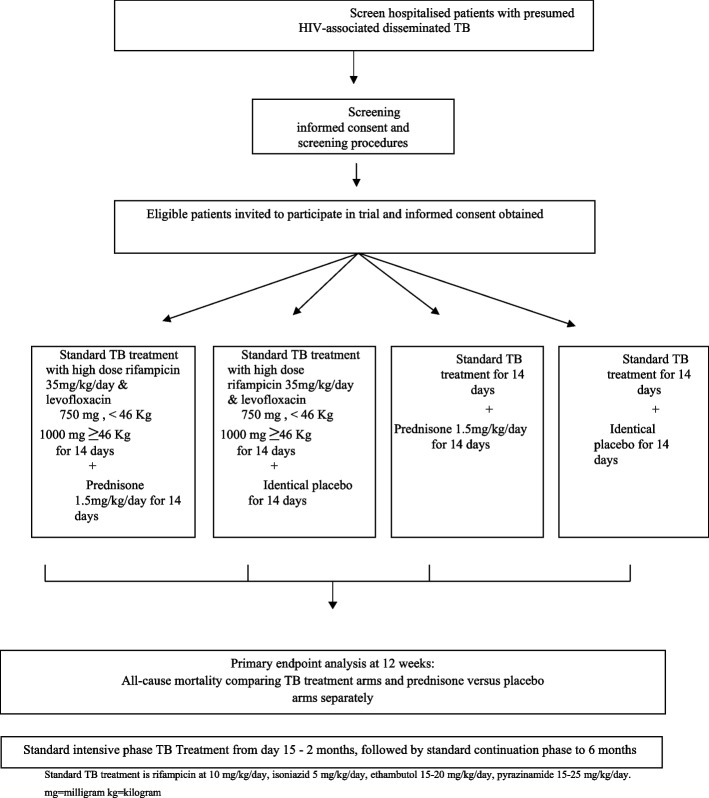
Trial schema. Standard TB treatment is rifampicin at 10 mg/kg/day, isoniazid 5 mg/kg/day, ethambutol 15–20 mg/kg/day, pyrazinamide 15–25 mg/kg/day. mg milligramme, kg kilogramme

The trial is a phase III, randomised controlled superiority trial, with a 2 × 2 factorial design enrolling patients hospitalised with HIV-associated disseminated tuberculosis. We are investigating the safety and efficacy of two interventions: [1] intensified tuberculosis treatment with higher dose rifampicin and the addition of levofloxacin and [2] adjunctive prednisone, both for the initial 14 days of tuberculosis treatment. The first intervention involves open-label standard weight-based tuberculosis treatment (rifampicin, isoniazid, ethambutol and pyrazinamide) with additional rifampicin to achieve a dose of 35 mg/kg daily for 14 days plus levofloxacin (750 mg for weight < 46 kg, 1000 mg for ≥ 46 kg) for 14 days and the comparator is standard weight-based four-drug tuberculosis treatment with rifampicin dosed at 10 mg/kg daily. The second intervention is placebo-controlled prednisone at 1.5 mg/kg/day for 14 days. All patients continue standard tuberculosis therapy from day 15 to complete 6 months of tuberculosis treatment Fig. 1.

## Methods: participants, interventions and outcomes

### Study setting {9}

The study is being conducted at three secondary-level public sector hospitals in Cape Town, South Africa, namely Mitchells Plain Hospital, Khayelitsha Hospital and New Somerset Hospital. Between 01 June 2022 and 22 June 2023, there were more than 50,000 people diagnosed and recorded with TB in the Western Cape province [55]: 4074 in the Mitchells Plain subdistrict, 3918 in the Khayelitsha subdistrict and 4578 in the Western subdistrict of Cape Town [55]. Khayelitsha Hospital is a 330-bed hospital [56] with 74 medical beds (personal communication). Mitchells Plain Hospital is a 390-bed hospital. It has about 120 medical beds (personal communication). New Somerset Hospital is a 352-bed hospital with 91 medical beds (personal communication).

### Eligibility criteria {10}

The inclusion and exclusion criteria are listed in Table 1. The exclusion criteria include conditions that may increase the risk of harm related to either of the interventions. Patients who require corticosteroids for an existing indication are also excluded.

**Table 1:** Eligibility criteria

**Inclusion criteria**	
**1. Aged ≥18 years**	
**2. Confirmed HIV infection**	
**3. Disseminated TB confirmed by one or more of the following tests being positive** **a. Lysed blood Xpert Ultra positive for MTB** **b. Concentrated urine Xpert Ultra positive for MTB** **c. Urine Alere LAM positive**	
**4. Hospital clinical team made decision to initiate TB treatment**	
**Exclusion criteria**	
**1. Pregnant or breastfeeding**	
**2. Current active SARS-CoV-2 infection (tested SARS-CoV-2 PCR positive on nasopharyngeal, mid-turbinate swab or sputum within 14 days prior to presentation).**	
**3. TB treatment within the last 1 month or more than 2 doses of TB treatment received during current illness**	
**4. Known rifampicin resistance**	
**5. Neurological TB including TB meningitis, tuberculomas/space-occupying lesions, cerebral oedema and TB spine**	
**6. Receiving or requiring corticosteroid or other immunosuppressive therapy**	
**7. Alanine transferase >120 IU/L or total bilirubin >34 μmol/L**	
**8. Plasma CrAg positive or cryptococcal meningitis**	
**9. Current malignancy requiring active treatment (including any Kaposi sarcoma lesions)**	
**10. Patients on PI-based antiretroviral therapy for longer than 2 years who do not have evidence of recent virological suppression (in the past 12 months)**	
**11. Diabetic ketoacidosis or hyperosmolar non-ketotic acidosis**	
**12. Uncontrolled epilepsy (known epileptic who is not currently taking treatment, known epileptic on treatment but had a seizure in the past one year)**	

### Who will take informed consent? {26a}

The doctors, nurses and clinical research workers have the responsibility of taking informed consent. After the consent process, an immediate review and quality control check is completed by another team member. Consent is taken in the patient’s language of choice.

Two consent processes occur prior to enrolment. A screening consent is completed at screening and an enrolment consent as appropriate thereafter. If a patient is unable to consent, the team attempts to get in touch with the next of kin or relative for consent. If the patient is unable to consent and the next of kin cannot be reached, they are entered into a delayed consent process described below.

### Delayed consent

We have approval from the University of Cape Town Human Research Ethics Committee to screen and enrol patients who are confused or have depressed level of consciousness, who are unable to provide informed consent themselves and have no next of kin available to provide consent. These patients should have documented HIV status and their ongoing participation, if enrolled, requires permission from an independent consent review committee that we have established (consisting of independent health care workers and community members). The trial team provides documentation of efforts made to contact next of kin and an assessment of capacity to consent daily, to the independent consent review committee, by day 5 of enrolment. If the committee agrees that reasonable efforts to contact family were made, they give a recommendation to the trial team to request the ethics committee for a consent waiver by day 7 of the trial. A consent waiver allows the trial team to keep the patient in the trial until either the patient can consent for themselves or a next of kin is found.

The rationale for including these patients is that these are the most severely ill group of patients with HIV-associated disseminated TB. Patients with disseminated TB often manifest a sepsis syndrome including altered mentation. These patients are severely ill and would potentially benefit the most should these interventions be found to be effective and safe. Studies often exclude severely ill patients but we contend that excluding the most severely ill patients could potentially create a treatment evidence gap amongst patients with HIV-associated disseminated TB. If we excluded this group of patients, this could also bias the findings and affect their generalisability. It was therefore important for us to develop procedures for including these severely ill patients whilst ensuring their autonomy is protected.

Once patients with reduced capacity to consent are enrolled, the study team checks daily if capacity has been regained by the participant and if next of kin can be reached for consent. If the participant is unable to consent and the next of kin is not contacted by day 5, the consent review committee is contacted for permission to retain the participant in the trial. The consent review committee are tasked with deciding whether the trial team has made reasonable efforts to contact the next of kin by day 7 of enrolment. If the team demonstrates reasonable efforts in having tried to contact the next of kin and there is evidence that daily review of the participant for regained capacity has occurred, the committee gives permission for the trial team to contact the ethics committee for a consent waiver for the participant to continue in the trial. If efforts demonstrated by the team are not adequate, the committee could recommend the participant be withdrawn from the study and all samples discarded.

Assessment of capacity to consent and efforts to contact next of kin are conducted daily, with the aim of obtaining informed consent as soon as is feasible. We obtain delayed consent from the participant whenever full capacity is regained if permission was given by a next of kin or a waiver of consent was obtained. See delayed consent process in the Appendix.

### Additional consent provisions for collection and use of participant data and biological specimens {26b}

We have ethical approval for the study from the University of Cape Town Faculty of Health Sciences Human Research Ethical Committee, HREC ref 001/2021. We also have permission from UCT Institutional Biosafety Committee (ref IBC053-2020) for the use of biological specimens. We obtain consent for sub-studies when the main study consent is taken, and patients have the option to take part in the main study without being part of the sub studies.

## Interventions

### Explanation for the choice of comparators {6b}

The trial is comparing the interventions to the standard of care for disseminated TB treatment in HIV. This standard of care is current practice and defined in national and international guidelines [57, 58].

## Interventions description {11a}

### Intervention 1: intensified tuberculosis treatment versus standard tuberculosis treatment

Participants in the control arm receive daily fixed dose combination tablets each containing isoniazid 75 mg, rifampicin 150 mg, ethambutol 275 mg and pyrazinamide 400 mg with the dose according to their weight band (Table 2) for 8 weeks. Those in the intervention arm receive the above standard treatment plus additional rifampicin to make the dose up to 35 mg/kg daily plus additional levofloxacin 750 mg or 1000 mg daily for 14 days. The study pharmacist prepares, packages and labels the medication in blister packs. The medication is supplied by the trial for the first 14 days and then by the routine treating facility from day 15 onwards. Following the first 14 days, participants from both arms complete the intensive and continuation phase weight-based standard tuberculosis treatment.

**Table 2:** Tuberculosis treatment for participants randomised to the control arm

Standard treatment of new and previously treated tuberculosis for adults in the South African National Department of Health Guidelines			
Pre-treatment body weight	Intensive phase (daily for 2 months)	Continuation phase (daily for 4 months)	
	RHZE (150/75/400/275)	RH (150/75)	RH (300/150)
30–37 kg	2 tablets	2 tablets	
38–54 kg	3 tablets	3 tablets	
55–70 kg	4 tablets		2 tablets
>70 kg	5 tablets		2 tablets

*R* rifampicin, *H* isoniazid, *Z* pyrazinamide, *E* ethambutol

#### Experimental arm: rifampicin dosing strategy

We are using a similar dosing strategy to that used in the LASER-TBM trial, a phase IIA trial of the safety and tolerability of increased dose rifampicin and adjunctive linezolid, with or without aspirin for HIV-associated tuberculous meningitis [59]. Simulations were performed to determine the dose of rifampicin required to achieve the most equitable drug exposures across the weight range 30 to 100 kg. Demographic data of a reference cohort of tuberculosis patients (*n* = 1225), with or without HIV co-infection, recruited in clinical trials conducted in West Africa and South Africa were used for the simulations [60–63]. An additional 12,250 virtual patients were generated using the weight and height distributions of the 1225 patients to increase the number of patients with a weight close to the boundaries of the weight range. Parameter estimates of the population PK model for rifampicin were used to simulate (100 replicates) rifampicin exposures [17]. This strategy approximates equitable exposures across weight bands to enable similar rifampicin exposures to be tested for all participants and to avoid lower exposures for patients in lower weight bands compared with higher weight bands when using the same milligramme per kilogramme dose.

Four dosing scenarios were evaluated using the weight-band based dosing with four-drug fixed drug combination (FDC) tablets and extra rifampicin tablets with each tablet containing 150 mg or 600 mg rifampicin. The FDC tablets were assumed to have 20% reduced bioavailability based on data from a clinical trial where the same formulation was used [64]. In this trial, we are using the same and similar FDC tablets to that reported in this publication that are provided by the Department of Health [61].

Table 3 shows the dosing in weight bands, providing balanced distribution in predicted exposures, which are being used to dose rifampicin in our trial in the experimental arm.

**Table 3:** Rifampicin dosing for participants randomised to the experimental arm

WHO	Band 1	Band 2	Band 3		Band 4
New-Strat TB trial	Band 1	Band 2	Band 3	Band 4	Band 5
**Weight range**	30–37 kg	38–54 kg	55–65 kg	66–70	> 70 kg
**R10HZE**	300	450	600	600	750
**R25 additional**	1200	1350	1500	1650	1950
**Total rifampicin**	1500	1800	2100	2250	2700
**Weight at 35 mg/kg dose**	43	51	60	64	77
**Dose at lower weight limit (mg/kg)**	50	47	38	41	38
**Dose at upper weight limit (mg/kg)**	41	33	32	35	30

### Intervention 2: corticosteroids versus identical placebo

The intervention is prednisone tablets (5 mg) and the control arm receives identical placebo tablets. The study medication has been manufactured and supplied by the Gulf Drug Company, Durban, South Africa. This generic pharmaceutical company supplies prednisone to public sector hospital pharmacies in South Africa (marketed as Trolic 5 mg tablets). Participants receive 1.5 mg/kg daily of prednisone (or identical placebo) for 14 days. Participants start this study medication on the same day as trial tuberculosis medication. Prednisone or placebo is stopped at study day 14. The prednisone dose is not weaned because of the short duration of therapy and because concomitant rifampicin induces prednisone metabolism resulting in predicted decreasing exposure over 14 days of prednisone administration (Tables 4 and 5).

**Table 4:** Levofloxacin dosing for participants randomised to the experimental arm

Weight	Levofloxacin dose
<46 kg	750 mg daily
≥46 kg	1000 mg daily

**Table 5:** Study medication dose adjustment for participants with renal dysfunction

Drug	eGFR 50–90	eGFR 30–50	eGFR 10–30	eGFR < 10	Dialysis
Rifampicin	No dose adjustment	No dose adjustment	No dose adjustment	No dose adjustment	No dose adjustment
Isoniazid	No dose adjustment	No dose adjustment	No dose adjustment	No dose adjustment	No dose adjustment
Ethambutol	15**–**25 mg/kg daily	15**–**25 mg/kg every 24**–**36 h	15**–**25 mg/kg every 36**–**48 h	15 mg/kg every 48 h	15 mg/kg every 48 h after dialysis for HD and PD. 15**–**25 mg/kg daily on CRRT
Pyrazinamide	25 mg/kg daily	25 mg/kg daily	25 mg/kg every 48 h	25 mg/kg every 48 h	25 mg/kg every 48 h after HD. 25 mg/kg every 24 h on PD/CRRT
Levofloxacin (patients <46 kg)	750 mg daily	750 mg every 48 h	750 mg every 48 h	750 mg every 48 h	750mg every 48 h
Levofloxacin (patients ≥46 kg)	1000 mg daily	1000 mg every 48 h	1000 mg every 48 h	1000 mg every 48 h	1000 mg every 48 h
Prednisone (or placebo)	No dose adjustment	No dose adjustment	No dose adjustment	No dose adjustment	No dose adjustment

*eGFR* estimated glomerular filtration rate, *HD* haemodialysis, *PD* peritoneal dialysis, *CRRT* continuous renal replacement therapy (table adapted from information obtained from Sanford guide App version 6.4.12, the EMGuidance application (https://emguidance.com) and levofloxacin doses as per Republic of South Africa: Department of Health: Management of Rifampicin resistant tuberculosis: A clinical reference: 2019

### Criteria for discontinuing or modifying allocated interventions {11b}

Patients are followed up until treatment completion at 6 months. There is more regular follow up in the first 14 days of therapy. Liver function tests, renal function and a full blood count are done on follow-up days 2, 4, 7 and 14. Significant hepatitis, or isolated hyperbilirubinemia with symptoms, results in a change to alternative TB treatment but the continuation of the prednisone/placebo. More details of this are described below.

As far as possible, the interventions are continued unless there are safety concerns. In this case, the drug that is likely to be the culprit is stopped and if needed an alternative TB treatment regimen prescribed.

If sepsis due to an infection other than TB develops, there is a new diagnosis of a malignancy or it is deemed for any reason that prednisone may place a participant at health risk, the prednisone/placebo study medication is discontinued.

### Strategies to improve adherence to interventions {11c}

Medication is packed by trial pharmacists in a clearly labelled blister pack. When patients are still hospitalised, medication is issued by trial nurses over the weekdays and hospital nurses over the weekend. Patients are counselled on possible side effects and encouraged to report any symptoms. The nurses teach the patients how to take the medication on discharge. Empty blister packs are returned to the trial team when medication is finished. Only 1 week’s TB treatment is issued at a time since patients are reviewed on day 7. They receive the second blister pack to complete 14 days at the day 7 review. Counselling is done to support adherence at all visits.

## Relevant concomitant care permitted or prohibited during the trial {11d}

### Clinical management

#### Antiretroviral therapy

South African National Department of Health guidelines are followed for ART [65]. All patients who fulfil criteria for entering this trial are eligible to start, restart or continue ART according to these guidelines. Patients start and continue ART at their local community health centres via referrals from the trial team or may be started on ART by the trial team at the study site. In patients who have interrupted ART or are ART naïve, ART initiation is timed according to the CD4 count. Participants with CD4 counts less or equal to 50 cells/mm^3^ are commenced on ART at 2 weeks on tuberculosis treatment [65]. Those with CD4 counts above 50 cells/mm^3^ start ART within 8 weeks of TB treatment. Participants initiating ART with CD4 counts below 100 cells/mm^3^ are started on concomitant prednisone (40 mg for 14 days then 20 mg for the next 14 days) for prevention of paradoxical TB-IRIS in line with findings of the PredART trial [40].

## Cotrimoxazole prophylaxis

Tuberculosis is an indication for cotrimoxazole prophylaxis in HIV-positive patients. All trial participants are eligible for co-trimoxazole prophylaxis unless they are known to have a sulphonamide allergy. Dapsone will be considered for those with sulphonamide allergy.

## Management of presumed paradoxical TB-IRIS

We are monitoring for paradoxical TB-IRIS. This includes patients who develop new or recurrent TB symptoms or fever, enlarging lymph nodes or worsening chest radiograph pulmonary infiltrates, enlarging effusions or new or recurrent neurological features. TB-IRIS is a diagnosis of exclusion and appropriate investigations to exclude other diagnoses are done.

If the diagnosis of paradoxical TB-IRIS is made with reference to the International Network for the Study of HIV-associated IRIS (INSHI) case definition [66, 67], corticosteroids may be prescribed to treat TB-IRIS at physician discretion.

## Management of drug-induced liver injury (DILI)

If drug-induced liver injury occurs during follow-up, patients are managed according to local clinical guidelines which are based on the American Thoracic Society guidelines for management of TB drug hepatotoxicity [68].

Elevation of alanine transaminase (ALT) of more than three times upper limit of normal with symptoms or ALT of more than five times with or without symptoms requires stopping of tuberculosis treatment. Patients are placed on at least three alternative drugs whilst monitoring for improvement of liver function tests. Drugs considered in the alternative regimen are linezolid, levofloxacin, terizidone, ethambutol, ethionamide and amikacin. Rechallenge of first-line drugs is considered when total bilirubin is normal and ALT <100 IU/l. Rifampicin is rechallenged first followed by isoniazid. Pyrazinamide is only rechallenged in certain patients. There is no rechallenge of high dose rifampicin in this trial; participants in the intensified tuberculosis treatment arm with DILI are rechallenged with standard TB treatment. Adjustments may be made to the ART regimen and cotrimoxazole prophylaxis may be stopped. The prednisone/placebo study medication is not stopped in event of a DILI.

## Management of isolated hyperbilirubinaemia

Rifampicin competes for the bilirubin transporter in the liver and through this mechanism may cause elevated bilirubin in the absence of liver injury. This occurs in the first 2 weeks of rifampicin treatment and tends to be self-limiting despite continuation of the drug. Patients who develop isolated bilirubin elevation, but remain asymptomatic without INR elevation, without elevation of ALT to more than three times upper limit of normal and without alkaline phosphatase elevation to more than three times baseline, will continue tuberculosis treatment with close monitoring of bilirubin and these parameters.

## Management of non-tuberculous mycobacterial (NTM) infections

If patients are found to have a disseminated non-tuberculous mycobacterial infection after enrolment, they are commenced on appropriate treatment and continue trial follow-up. In such patients who were urine LAM positive, but all tests for tuberculosis were negative (including both the blood and urine Xpert MTB/RIF Ultra), a decision to stop tuberculosis treatment may be made after full clinical review.

## Rifampicin-resistant tuberculosis

Known rifampicin resistance is an exclusion criterion, but if the diagnosis is made after enrolment patients are switched to appropriate rifampicin-resistant tuberculosis treatment based according to national guidelines. Participants diagnosed with rifampicin-resistant TB continue to be followed up in the trial.

## Isoniazid-resistant tuberculosis

This is diagnosed on drug susceptibility testing performed on culture isolates and is treated according to national guidelines with the addition of levofloxacin to the standard first-line tuberculosis drug regimen (RHEZ) for the remaining duration of treatment. These patients are also followed up to trial completion.

## New onset seizures

If patients develop new seizures in the first 2 weeks of the trial and are in the intensified TB treatment arm and thus receiving high dose rifampicin and levofloxacin as part of their TB treatment, levofloxacin is stopped due to its potential to reduce seizure threshold [69] but the high dose rifampicin and the other TB drugs are continued.

## Concomitant medication

### Protease inhibitors

Co-administration of protease inhibitors (PIs) and rifampicin results in markedly reduced PI concentrations [70] . Patients on PI-based ART regimen for more than 2 years who do not have evidence of recent viral suppression are not eligible for the study. Patients who have been on PI-based ART for less than 2 years and those with virological suppression on PI-based ART are switched to a dolutegravir-based regimen and are eligible for enrolment.

## Non-steroidal anti-inflammatory drugs (NSAIDs)

The use of NSAIDs is prohibited whilst on study intervention (for the first 14 days) to avoid the potential adverse effects of gastritis and peptic ulcers, which can be further aggravated by concomitant prednisone administration.

## Drugs that potentially prolong QT

The effect of levofloxacin on QT is minimal [32]; therefore, other QT prolonging drugs are not prohibited.

### Provisions for post-trial care {30}

This is not applicable as the interventions are only in the first 2 weeks of TB therapy.

### Outcomes {12}

The primary efficacy endpoint is all-cause mortality at 12 weeks.

Secondary endpoints:

1. In-hospital mortality during index admission.
2. All-cause mortality at 2 and 24 weeks, respectively.
3. Change in venous lactate over 14 days of study medication administration.
4. Change in C-reactive protein over 14 days of study medication administration.
5. Change in haemoglobin over 14 days of study medication administration.

Safety and tolerability endpoints:

1. Occurrence of hepatotoxicity using the American Thoracic Society (ATS) hepatotoxicity criteria: alanine aminotransferase (ALT) elevation of more than three times the upper limit of normal (ULN) in the presence of hepatitis symptoms and/or jaundice or five times the upper limit of normal in the absence or presence of symptoms.
2. Corticosteroid-associated adverse events classified by severity and relation to study drug and will be reported if these develop within the 12 weeks of enrolment.
3. Laboratory safety data (grade 3 and 4 abnormalities using the ACTG grading system): liver function tests (alanine and aspartate aminotransferase (ALT, AST), alkaline phosphatase (ALP), International Normalized Ratio (INR), conjugated and total bilirubin (CBR, TBR)), glucose, full blood counts (including white cell, neutrophil and platelet counts plus haemoglobin) and electrolytes (sodium, potassium) and creatinine.
4. Occurrence of other opportunistic infections (AIDS-related, bacterial, fungal and viral) and malignancies (Kaposi’s sarcoma) up to 12 weeks.
5. Occurrence of paradoxical tuberculosis immune reconstitution inflammatory syndrome (TB-IRIS) in patients starting ART up to 12 weeks.
6. All grade 3 and 4 clinical adverse events (using the ACTG grading system)
7. Serious adverse events
8. Adverse events requiring study drug interruption and or withdrawal
9. Adverse drug reactions attributed to study drug

### Participant timeline {13}

#### Study visits

Follow-up visits occur in the ward whilst the participant remains admitted and then in the outpatient department after discharge from hospital. The following visits are scheduled: enrolment (day 1), day 2, day 4, day 7, day 14, week 4, week 12 and week 24. Visits on days 2, 4, 7 and 14 have a 2-day window period (1 day earlier or 1 day later) to complete visits. Visits at weeks 4, 12 and 24 have a window of 1 week earlier or 1 week later. Telephonic follow-up and the use of electronic databases is acceptable to ascertain treatment progress, episodes of clinical deterioration and vital status from week 4 visit onwards if the patient is unable to attend visits in person or if a patient is not contactable. Women of childbearing age are advised at discharge to use effective (barrier) contraception and consider abstinence whilst on study medication to day 14.

Outside of scheduled visits, participants are free to attend for an unscheduled visit if they feel unwell especially in the first 2 weeks after the enrolment. At discharge from hospital, participants are followed up by the trial team in the outpatient department for the first 14 days. They are then referred to their local clinic and followed up for HIV and tuberculosis treatment as per local guidelines, with subsequent review by the clinical trial team at weeks 4, 12 and 24 in the outpatient department.

Cumulative mortality at 12 weeks is ascertained using available medical records, hospital notes, death certificates, clinical notes, electronic databases, death register and history from family members or other contact persons. We endeavour to gather sufficient clinical details to facilitate assessment of the most probable cause of death. Causes for clinical deterioration are ascertained during the first 12 weeks of follow-up.

### Loss to follow-up

Participants are classified as lost to follow-up if the trial team fails to make contact on least two consecutive planned visits and find no evidence that the patient accessed health care and no evidence that they died (by reviewing electronic health care databases and the national death register) at the end of the participant’s follow-up period.

**Table:** Study procedures and timeline

**Procedures**	**D1**	**D2**	**D4**	**D7**	**D14**	**D28**	**Wk12**	**Wk24**
Informed Consent^a^	X							
Randomization	X							
Initiation of study medication	X							
Medical History	X							
Current symptoms	X		X	X	X	X	X	X
Physical Examination	X		X	X	X	X	X	X
Adverse Events			X	X	X	X	X	X
Medication review	X		X	X	X	X	X	X
Pill count			X	X	X	X	X	X
ECOG score	X	X	X	X	X	X	X	
qSofa	X	X	X	X	X	X	X	
AVPU	X	X	X	X	X	X	X	
Report of clinical deterioration			X	X	X	X	X	X
Nose, groin, rectal swab & stool collection	X		X	X				
Urea/Creatinine & Electrolytes		X	X	X	X			
Glucose		X	X	X	X	X		
Full Blood Count and differential		X		X	X			
LFTs (ALT, AST, ALP, TBR)		X	X	X	X	X		
CRP		X	X	X	X	X		
Venous lactate		X	X	X	X			
CD4, HIV viral load		X						
Mycobacterial blood culture^b^								
Immunology assays		X	X	X	X			
Storage blood		X	X	X	X			
Storage urine		X	X	X	X			
RNA storage		X	X	X	X			
DNA storage		X						

Participants must not have had >2 doses of TB medication before initiation of study medication

^a^Informed consent can be repeated or revisited at any of the follow up visits as needed

^b^Performed at screening for all patients and repeated only for sub-study patients on day 2, 4, 7 and 14

*ECOG* Eastern Cooperative Oncology Group, *qSOFA* quick Sequential Organ Failure Assessment score, *AVPU* Alert, Verbal, Pain, Unresponsive, *diff* differential cell count, *LFT* liver function tests, *ALT* alanine transaminase, *AST* aspartate aminotransferase, *ALP* alkaline phosphatase, *TBR* total bilirubin, *CD4* cluster of differentiation 4, *RNA* ribonucleic acid, *DNA* deoxyribonucleic acid, *CRP* C-reactive protein

### Sample size {14}

The study is designed to demonstrate superiority of each intervention separately over the standard of care with a *p*-value threshold of 0.05 used to determine a significant result. There is no adjustment for multiple comparisons. We assume mortality at 12 weeks to be 28% in the standard of care arm. This was based on extrapolation of mortality data from previous study amongst patients with HIV associated TB [4]. The trial is powered to demonstrate a relative reduction in mortality of 32% (reduction from 28 to 19% in absolute 12-week mortality). To have adequate power of 80% and alpha 0.05, a sample size of 347 per arm is required. Inflating this number to account for 5% loss to follow-up, we will recruit 732 participants to the trial. The factorial design of the trial may result in interaction between the interventions, and this will be evaluated as a secondary endpoint. This interaction analysis is relatively underpowered, and we would only be able to detect a large interaction.

### Recruitment {15}

HIV-positive adults admitted to hospital with untreated, HIV-associated disseminated TB, diagnosed by one or more of the three disseminated tuberculosis tests we are using (urine Alere LAM assay, urine Xpert MTB/RIF Ultra, and blood Xpert MTB/RIF Ultra) and fulfilling other enrolment criteria are enrolled into the trial after obtaining informed consent.

Eligible patients are identified by members of the study team from the post intake ward rounds in the emergency department of recruitment hospitals every morning as well as by screening through the hospital admission book or through patient folders in the emergency department and the medical wards. Hospital staff are aware of the trial and refer appropriate patients directly to the study team. Patients are invited to be screened for participation in the clinical trial if they are deemed eligible. Patients are provided with a screening informed consent form in the language of their choice (Afrikaans, Xhosa or English). Patients are given time to read the form or it is read to them by a study team member or family member. All trial information sheets and consent forms are available electronically and in paper form.

### Assignment of interventions: random allocation

#### Sequence generation {16a}

A randomisation list has been generated using a 1:1:1:1 ratio, permuted block randomisation with variable block sizes and is stratified by study site. The randomisation list has been uploaded to REDCap. On randomisation, REDCap uses the randomisation list and study site field to allocate the participant to the next available treatment arm assignment for that site. This ensures randomisation is stratified by recruitment site; no other stratification occurs.

#### Concealment mechanism {16b}

The randomisation sequence uploaded into REDCap is only accessible to the data manager and cannot be edited. The randomisation list is inaccessible to clinical staff. To ensure allocation concealment treatment arms are only allocated after the time of enrolment and are accessible to study staff as follows: after randomisation to TB treatment arm, the allocation for each participant is provided to the clinical staff and trial pharmacists. This is visible in REDCap after randomisation. The allocation to prednisone or placebo is provided only to the trial pharmacist and is not visible to the clinical staff.

#### Implementation {16c}

The allocation sequence was generated by the randomisation consultant. The trial pharmacist assigns study medication based on random allocation of that participant and if unable to access random allocation on REDCap for any reason the randomisation consultant has access to the back up and is contacted.

### Assignment of interventions: blinding

#### Who will be blinded {17a}

The trial team is not blind to the TB treatment allocation but is blinded to the prednisone/placebo allocation. The trial pharmacist is unblinded to both study interventions.

#### Procedure for unblinding if needed {17b}

The first intervention is open label and there is thus no need for unblinding.

For the second intervention (prednisone or placebo), emergency unblinding may be done in the following situations:

- Severe adverse events that may be related to the use of corticosteroids, e.g. severe psychosis, new diagnosis of malignancy especially Kaposi’s sarcoma
- Development of new SARS-CoV-2 infection during the first 14 days of the trial
- Any other clinical occurrence where the investigator deems it necessary for patient safety.

The emergency unblinding procedure involves the investigator verifying with the PIs that unblinding is needed. The study pharmacist is then contacted. If the study pharmacist is not available, the data manager with access to the randomisation code on REDCap will be contacted. This will be documented on an emergency unblinding case report forms (CRFs).

### Data collection and management

#### Plans for assessment and collection of outcomes {18a}

For this trial, electronic CRFs completed online in real time are considered the main source documentation and are completed by delegated staff members. These electronic CRFs are used to collect demographic, baseline and follow-up clinical data including laboratory data. The laboratory result report constitutes the laboratory source document. When the laboratory result is received or viewed, the study doctor enters results in the Laboratory Results CRF in real time and uploads a de-identified copy of the result onto the electronic database and makes a note of actions to be taken for any abnormal result. The study team member entering this checks that the data on the source document and the electronic CRF correspond, and another team member does a quality control check as per our Clinical Quality Management Plan.

#### Plans to promote participant retention and complete follow-up {18b}

The study has community research workers who counsel, educate and support participants with regard to the trial and medication adherence. Telephonic visits are acceptable from day 28 visit should this be preferred by the participant. Our nurses make telephonic contact with patients before appointment dates to remind them of the visits. Patients have access to trial phone numbers for questions and medical advice if required.

#### Data management {19}

The investigators have developed a Clinical Quality Management Plan (CQMP) to ensure high quality and reliable data. The electronic data collection tool in REDCap has been designed in such a way that range checks for values are pre-entered and will pop up a warning when an out-of-range value is entered. Access to the REDCap database is password protected with each member having their own username and password.

#### Confidentiality {27}

REDCap is a secure web application for building and managing online surveys and databases.

The platform implements appropriate administrative, physical and technical safeguards to ensure the confidentiality, integrity and security of electronic health information. All trial data is de-identified and coded with a study number. Documentation, data and all other information that relates to individual patients is held in strict confidence. No information concerning the patient will be released to an unauthorised third party, without written approval of the participant except as necessary for trial monitoring or regulatory review. University of Cape Town Human Research Ethics Committee (UCT HREC) and South African Health Products Regulatory Authority (SAHPRA) are authorised third parties.

#### Plans for collection, laboratory evaluation and storage of biological specimens for genetic or molecular analysis in this trial and future use {33}

As part of the consent process, patients are requested to separately provide permission for storage of their samples for future studies including genetics. Ethical approval will be sought at the stage where specific research studies beyond those included in the protocol are planned using these samples. At screening, days 2, 4, 7 and 14, blood and urine are stored.

### Statistical methods

#### Statistical methods for primary and secondary outcomes {20a}

The number of patients screened and enrolled or excluded will be summarised and reasons for exclusion listed. The number of participants who discontinue or are lost to follow-up will be tabulated by reason and time point. This information will be summarised in a CONSORT flow diagram. Patients in each treatment arm will be described with respect to baseline characteristics. Medians and interquartile ranges will be used as measures of central tendency for continuous characteristics and counts and percentages for categorical characteristics. The potential clinical importance of any differences between arms will be noted but statistical tests of significance will not be undertaken to compare arms.

#### Primary endpoint analysis

For analysis of the primary endpoint, we will present the 12-week mortality by treatment arm. We will include all participants who were randomised and received at least one dose of study medication for that treatment arm in a modified intention-to-treat analysis evaluating the efficacy of the two interventions. The efficacy of intensified TB treatment to reduce 12-week mortality will be tested by comparing the proportion of participants who died in the group who received intensified tuberculosis treatment and the group who received standard tuberculosis treatment. The efficacy of prednisone to reduce 12-week mortality will be tested by comparing the proportion of participants who died between the arm who received prednisone compared to those who received placebo. The primary statistical analyses will be performed using the Chi-squared test. As secondary analyses, time to event analyses will be performed using Cox proportional hazards models or an accelerated failure time model (as appropriate) which will be adjusted for the other intervention and pre-specified clinical variables, such as age, sex and HIV viral load. These variables will be included in the statistical analysis plan. Adjusted estimates will be presented with 95% confidence intervals. Loss to follow-up will likely be low as we have been able to ascertain vital status during follow-up in >95% of HIV-positive inpatients enrolled in previous studies in Cape Town. We will report the number of participants who are lost to follow-up and will do a sensitivity analysis to ascertain if they are systematically different from the rest of the cohort. In the primary analyses (using Chi-squared test) participants lost to follow-up will be assumed to be alive at week 12. We will include participants who are lost to follow-up in the time to event analyses and censor patients at the last date of contact with the clinical team or other health care services.

#### Secondary endpoint analyses

Secondary per protocol analyses will be performed which will exclude participants who stopped protocol defined treatment. Patients with drug toxicity who interrupt protocol defined treatment and are later rechallenged onto tuberculosis treatment will also be excluded in these analyses. Secondary endpoints will be analysed comparing study arms using the Chi-squared test (for categorical data) or Wilcoxon rank sum test (for continuous data). Time to event analyses (constructing Kaplan–Meier survival curves and compared using the rank sum tests, or Cox proportional hazards models or accelerated failure time models) will also be performed.

Interaction between the treatment interventions will be estimated and reported, even though the analyses are not formally powered to detect interaction.

#### Interim analyses {21b}

A data safety and monitoring board (DSMB) has been established with five members independent from the trial. Every 6 months, a member of the trial team and the trial statistician prepare an open efficacy and safety report for the committee with data aggregated across all participants, but not by treatment allocation. The trial statistician prepares a separate closed report with data presented by trial arm that is assessed by the DSMB to determine if the trial should be stopped, continued or modified. The clinical trial team is not privy to the closed report and has no access to interim results by study arm.

#### Methods for additional analyses (e.g. subgroup analyses) {20b}

Prespecified subgroup analyses of the primary endpoint will include stratification by:

- *Mycobacterium tuberculosis* blood stream infection (diagnosed by blood Xpert MTB/RIF Ultra assay and/or mycobacterial blood culture) or no blood stream infection
- Patients with CD4 cell count ≤100 cells/mm^3^ or > 100 cells/mm^3^
- Patients with haemoglobin ≤7 g/dL or >7 g/dL

#### Safety analyses

We will compare serious adverse events, grade 3 and 4 adverse events, all adverse events leading to study drug discontinuation and adverse drug reactions by study arm in two 2-way comparisons and by combined interventions in a 4-way comparison. This will be done using Chi-squared test without correction for multiple comparisons.

A statistical analysis plan will be finalised and approved by the Trial Steering Committee before the trial database is locked and unblinded.

Even though the TB treatment randomisation is open label, the investigators will deliberately not analyse or view the end point data in aggregate form during the trial other than in the closed report prepared for the Data and Safety Monitoring Board that the investigators will not see.

#### Methods in analysis to handle protocol non-adherence and any statistical methods to handle missing data {20c}

Modified intention-to-treat analyses will be used for the primary and secondary endpoint analyses, but per protocol analyses will also be performed as secondary analyses to account for those participants who do not receive the interventions as specified in the protocol. Missing data will be noted in the study publication and will be handled using multiple imputation where applicable.

#### Plans to give access to the full protocol, participant level data and statistical code {31c}

We will make the full protocol available on-line with the final trial publication which will be made open access. Anonymised participant level-data and statistical code will be shared upon request to investigators and after ethical approval.

### Oversight and monitoring

#### Composition of the coordinating centre and Trial Steering Committee {5d}

The trial has a coordinating committee composed of the principal investigators, lead investigator and study coordinator. This team ensures the day-to-day operations of the trial run smoothly. The principal investigators and lead investigator meet monthly to discuss the trial.

### Trial Steering Committee

The conduct of the clinical trial is overseen by the Trial Steering Committee (TSC), which includes the principal investigators, the lead investigator, senior clinicians from participating hospital and two independent members. The TSC meets every 4 months via a conference call. The TSC reviewed preparation for the trial and reviews clinical enrolment and adverse events on the study, analysis and sub-studies.

### Composition of the Data Safety and Monitoring Board Committee, its role and reporting structure {21a}

Oversight of the trial is provided by an independent Data Safety and Monitoring Board Committee (DSMB). This DSMB has five members: four independent and experienced HIV-associated TB researchers and an independent trial statistician. The DSMB charter specifies the procedures to be followed, the data to be reviewed by the DSMB and provides guidance for stopping based on harm and efficacy considerations. Open and closed statistical reports are prepared for the DSMB by trial statisticians. The first data meeting and safety analysis occurred, as planned, after 40 participants had been randomised. Subsequent meetings are 6 monthly with review of both safety and efficacy. The DSMB may decide to convene an unscheduled DSMB review if warranted by safety or data quality concerns. The DSMB is independent from the sponsor, the University of Cape Town.

### Adverse event reporting and harms {22}

In the event of an occurrence of a grade 3 and 4 adverse event or adverse drug reaction, the event as well as action taken will be recorded. The severity will be graded using the Division of AIDS (DAIDS) Table for Grading the Severity of Adult and Pediatric Adverse Events, Corrected Version 2.1, July 2017. All suspected unexpected serious adverse events (SUSARs), serious adverse events (SAEs) and deaths on the trial are reported to UCT HREC and SAHPRA. Annual (UCT HREC) and 6-monthly (SAHPRA) reports are submitted as per requirements from these bodies.

### Frequency and plans for auditing trial conduct {23}

The University of Cape Town as the sponsor has subcontracted monitoring of the trial to a clinical trials monitor with extensive experience of clinical trials monitoring in South Africa. The monitor is independent of the investigator team. The monitor performed a site initiation visit prior to initiation of recruitment and monitors participants’ data on REDCap according to a pre-specified monitoring plan. A monitoring visit occurred after enrolment of the first five participants and now occurs roughly quarterly depending on recruitment rate. The last monitoring visit will occur 1–2 days after database lock.

### Plans for communicating important protocol amendments to relevant parties {25}

Any protocol amendment is submitted to HREC and SAHPRA for approval before being implemented.

### Dissemination plans {31a}

The trial results will be published in a medical journal and will be made open access. We plan to obtain permission from our ethics committee prior to making the data available in an open data repository. This will be after trial completion, database lock and analyses completion. Findings will be presented at an international HIV conference, at local academic meetings and to the staff at the hospitals where the trial is being conducted. There are no publication restrictions.

### Sub-studies

There are several sub-studies nested within the trial. Patients may take part in the main trial without participating in the sub-studies. The consent process allows for participants to opt out of specific sub-studies. Sub-studies include:

1. *Pharmacokinetic sub-study* evaluating rifampicin concentrations on day 2 of the trial and their relationship to adverse events and bilirubin concentrations.
2. *Mycobacterium tuberculosis biomarkers sub-study* evaluating changes in quantitative markers of disseminated TB load over the 14 days of study medication comparing intensive and standard TB treatment arms. Criteria for this substudy includes having at least one positive Xpert Ultra at screening and a haemoglobin concentration of at least 6 g/dl without having had any TB treatment prior to enrolment. Biomarkers being evaluated are urine lipoarabinomannan (LAM), urine Xpert Ultra, blood Xpert Ultra and mycobacterial blood culture at screening, days 2, 4, 7 and 14. Semiquantitative concentration of LAM in urine using grade on the Alere LAM card, cycle threshold (Ct values) for blood and urine Xpert Ultra and time to positivity of blood culture will be used as measures of mycobacterial load. In a select group of patients who are blood Xpert Ultra positive, have a haemoglobin level of at least 6 g/dl and have had no more than one dose of TB treatment, DMN-Tre microscopy on lysed blood and quantification of neutrophil extracellular trap formation will be done at the same time points (screening, days 2, 4, 7 and 14).
3. *Immunology sub-study* which will assess host soluble inflammatory mediator concentrations in plasma (using Luminex) over the first 14 days comparing longitudinal changes between arms. Specifically, we will investigate whether trial interventions modulate the innate immune profile that was associated with mortality in our previous observational cohort study [5]. Another immunology sub-study will evaluate changes in transcriptomic profile on treatment using RNAseq performed on whole blood to assess whether the interventions alter the transcriptomic profile towards a profile associated with survival. We will also evaluate markers implicated in immunothrombosis and neutrophil extracellular trap (NET) formation in the blood and their relationship with *Mycobacterium tuberculosis* blood stream infection, trial interventions and outcomes.
4. *Limited post-mortem sub-study* to assess causes of death and better understand pathophysiology of HIV-associated disseminated TB. Minimally invasive autopsies using Trucut needle biopsies of organs including the lung, liver, spleen, heart, lymph nodes and bone marrow are performed after appropriate consent. Blood and cerebrospinal fluid are also collected. Microbiological and histological tests are performed on samples.
5. *Bacteriology sub-studies* evaluating changes in the gastro-intestinal microbiota, MRSA colonisation using groin and nasal swabs and changes in gastro-intestinal microbial resistance patterns on study medication.

## Discussion

The NewStrat-TB trial will provide evidence for whether intensified TB treatment with high dose rifampicin at 35 mg/kg and levofloxacin added to standard TB treatment for the first 2 weeks reduces mortality in patients hospitalised with disseminated HIV-associated TB. It will also provide evidence for whether adjunctive prednisone dosed at 1.5 mg/kg/day for 2 weeks reduces mortality in this patient population.

Patients with HIV-associated disseminated TB have a severe disease phenotype characterised by organ failure, sepsis and a dysregulated inflammatory response [4]. This places these patients at a high mortality risk. Amongst hospitalised patients, deaths tend to occur early despite the initiation of TB treatment [4]. Disseminated TB is currently treated with the same drug regimens and doses as patients with other more limited forms of TB such as pulmonary TB. We reason, based on our previous research, that these patients are often critically ill and more intensified and specific treatment strategies need to be evaluated to improve their survival.

There are many factors contributing to mortality of patients with HIV and tuberculosis. Late diagnosis of HIV, disengagement from HIV care and delays to TB diagnosis and treatment all play a role [1, 71, 72]. There are ongoing public health interventions and research efforts to address these factors. Routine HIV counselling and testing and universal test and treat strategy have improved early diagnosis and treatment of HIV. TB symptom screening and improved TB diagnostics, such as urine LAM and the Xpert MTB/RIF assay, have facilitated improved TB diagnosis and reduced delays to treatment [73–77]. Despite early diagnosis and treatment initiation for HIV-associated TB at a programme level, an unacceptably high number of patients still die early after starting TB treatment, particularly amongst those patients ill enough to require hospital admission [4, 6].

A major focus of current TB treatment research is justifiably focussed on treatment shortening and optimising the treatment of drug-resistant TB [78–80]. In addition to these priorities, it is important to focus treatment research on severe forms of TB that contribute disproportionately to global TB mortality and may need specific and targeted management approaches, such as disseminated TB and TB meningitis. The development of novel strategies that improve treatment outcomes for patients with HIV-associated disseminated TB could have major public health benefits, given the frequency of this condition in countries with a high co-prevalence of HIV and TB across the world. The results of this trial could potentially provide evidence for updates to guidelines for treatment of HIV-associated TB.

## Trial status

Most recent protocol is version 1.5, dated 22 February 2023. The trial began enrolment on 11 August 2021. We project that recruitment will be completed in the fourth quarter of 2024.

### Supplementary Information

**Supplementary Material 1.**

## Abbreviations

ACTG: AIDS clinical trials group
Ag: Antigen
AUC: Area under the curve
AIDS: Acquired immunodeficiency syndrome
ALT: Alanine transaminase
ALP: Alkaline phosphatase
ART: Antiretroviral therapy
AVPU: Alert voice pain unresponsive
aHR: Adjusted hazard ratio
CBR: Conjugated bilirubin
CRP: C-reactive protein
CRF: Case report form
CRRT: Continuous renal replacement therapy
CI: Confidence interval
Cmax: Maximum concentration
CQMP: Clinical quality management plan
DILI: Drug-induced liver injury
DNA: Deoxyribonucleic acid
DMN-Tre: 4-N,N-Dimethylamino-1,8-naphthalimide conjugate of trehalose
DSMB: Data safety and monitoring board
EBA: Early bactericidal activity
EMB: Ethambutol
ECOG: Eastern cooperative oncology group
eGFR: Estimated glomerular filtration rate
EM: Emergency
FBC: Full blood count
FDC: Fixed dose combination
FGF: Fibroblast growth factor
g/dl: Grammes per decilitre
H: Isoniazid
HD: Haemodialysis
HIV: Human immunodeficiency virus
HREC: Human research ethics committee
IRIS: Immune reconstitution inflammatory syndrome
INR: International normalized ratio
INSHI: International network for the study of HIV associated IRIS
IL: Interleukin
IFN-δ: Interferon gamma
Kg: Kilogramme
LAM: Lipoarabinomannan
LFTs: Liver function tests
MIC: Minimum inhibitory concentration
MIP: Macrophage inflammatory protein
MTB: *Mycobacterium tuberculosis*
MTBBSI: *Mycobacterium tuberculosis* blood stream Infection
Mg: Milligramme
NETS: Neutrophil extracellular traps
NTM: Non-tuberculous mycobacteria
NSAIDS: Non-steroidal anti-inflammatory drugs
PK: Pharmacokinetics
PD: Pharmacodynamics
PDGF: Platelet-derived growth factor
P.I: Protease inhibitors
PJP: *Pneumocystis jiroveci* pneumonia
qSOFA: quick Sepsis-related organ failure assessment
RNA: Ribonucleic acid
RNA seq: Ribonucleic acid sequencing
RR: Relative risk
R: Rifampicin
RIF: Rifampicin
SOP: Standard operating procedure
SAE: Severe adverse event
SUSAR: Suspected unexpected severe adverse event
SARS-Cov-2: Severe acute respiratory syndrome coronavirus 2
SAHPRA: South african health products regulatory authority
TGF: Transforming growth factor beta
TTP: Time to positivity
TSC: Trial steering committee
TBR: Total bilirubin
TB: Tuberculosis
UCT: University of cape town
Z: Pyrazinamide
